# The correlation between plasma lactoferrin and inflammatory biomarkers in type 2 diabetes with dry eye disease patients

**DOI:** 10.3389/fmed.2025.1569690

**Published:** 2025-07-28

**Authors:** Amani Y. Alhalwani, Shatha Jambi, Husain Alalgum, Hawazen Zarif, Sarah Alshareef, Abrar Babgi, Rawiah Alsiary, Faisal F. Alamri, Nizar Gusti, Salwa Alaidarous

**Affiliations:** ^1^College of Science and Health Professions, King Saud Bin Abdulaziz University for Health Sciences, Jeddah, Saudi Arabia; ^2^Department of Biomedical Research, King Abdullah International Medical Research Centre, Jeddah, Saudi Arabia; ^3^College of Applied Medical Sciences, King Saud bin Abdulaziz University for Health Sciences, Jeddah, Saudi Arabia; ^4^College of Medicine, King Saud bin Abdulaziz University for Health Sciences, Jeddah, Saudi Arabia; ^5^National Guard Health Affairs, Jeddah, Saudi Arabia; ^6^Department of Biochemistry, King Abdulaziz University, Jeddah, Saudi Arabia

**Keywords:** lactoferrin (LF), neutrophiles, enzyme-linked immunosorbent assay (ELISA), dry eye disease (DED), inflammation, type 2 diabetes (T2D), neutrophil to lymphocyte ratio (NLR), lactoferrin to neutrophil ratio (LFNR)

## Abstract

**Background:**

Lactoferrin (LF) is a primarily protein derived from the degranulation of neutrophils in plasma, and has been identified as a potential biomarker for dry eye disease (DED) and type 2 diabetes patients (T2D). This study aims to investigate the correlation between plasma lactoferrin and other inflammatory biomarkers, such as lactoferrin to neutrophil ratio (LFNR) and glycosylated hemoglobin A1c (HbA1C), in type 2 diabetic patients with dry eye disease (T2D-DED).

**Method:**

This study was conducted at the Diabetes Center outpatient clinics at King Abdulaziz Medical City, Jeddah, Saudi Arabia. The study included two groups: 26 healthy individuals and 41 T2D-DED patients. The plasma samples were collected and analyzed in the hospital for laboratory routine tests for neutrophil, lymphocyte, C-reactive protein (CRP), glycosylated hemoglobin A1c (HbA1C), and albumin (ALB), and data were collected retrospectively from hospital medical records. The same plasma samples were tested for LF using an in-house enzyme-linked immunosorbent assay (ELISA). The plasma lactoferrin to neutrophil ratio (LFNR) and neutrophil to lymphocyte ratio (NLR) were calculated. All statistical analyses were performed using PRISM software, with a *p*-value < 0.05 were considered significant.

**Results:**

LF concentrations were found to be 1.10 ± 1.0 μgmL^−1^ in T2D-DED patients and 0.5 ± 0.4 μgmL^−1^ in healthy individuals. Inflammatory biomarkers, LF, CRP, HbA1C, and LFNR, showed elevated levels in patients with T2D-DED, with statistically significant differences groups compared to healthy individuals. Additionally, there was a significant positive correlation in T2D-DED patients between LF with LFNR in T2D-DED patients (*p* = 0.0001) and HbA1C with LF (*p* = 0.035).

**Conclusion:**

The study indicated that LF levels and other inflammatory biomarkers are elevated in patients with T2D-DED. There are significant positive correlations between LF and HbA1C, as well as LF and LFNR in T2D-DED patients, which differ from the correlations found in healthy individuals. This suggests that the diagnostic and prognostic relevance of these biomarkers depends on whether the disease is present.

## 1 Introduction

Lactoferrin (LF) is an iron-binding glycoprotein of the transferrin family expressed and secreted in many bodily fluids, including milk, tears, and saliva (1). LF is also a prominent component of the secondary granules of neutrophils and is released in infected tissues and blood during the inflammatory process. LF has multiple functions, and it is considered a key component in the host’s first line of defense, as it can respond to various physiological and environmental changes. Besides its direct effects on host defense against bacteria, fungi, and parasites, possible roles in the modulation of the immune response were reported (2). The structural characteristics of LF provide functionality in addition to the Fe^3+^ homeostasis function common to all transferrins: strong antimicrobial activity against a broad spectrum of anti-inflammatory, and several functions (3, 4). LF controls the iron levels in cells and protects the body’s system (5). Additionally, the iron-binding function of LF plays a role in antioxidant activity, delaying the progression of oxidative stress-related disease and improving the quality of life (6). These properties were illustrated by many *in vitro* and *in vivo* experiments carried out on humans and animals (7–14).

The inflammatory status is critical for lactoferrin’s therapeutic action, mediated by numerous inflammatory indicators such as cytokines. A prior study examined LF as a diabetic treatment by monitoring pro-inflammatory cytokines like IL-6 (Interleukin-6) and TNFα (Tumor Necrosis Factor alpha) (15, 16). Mayeur et al. investigated plasma LF and other biomarkers in obese women, including lipid profile and glucose homeostasis biomarkers (17). They also found a significant increase in these biomarkers for obese diabetic women, with a significant correlation between plasma LF and plasma insulin when compared to obese non-diabetic women (17). Given this anti-inflammatory effect, insulin production increased while glycemia decreased significantly. These encouraging findings suggest that LF supplementation may be necessary for restoring immune balance and blood sugar levels in type 2 diabetes (T2D) patients.

T2D has been studied to expose dysfunction in the tear film and cause ocular diseases, including retinopathy (18) and DED (19, 20). The major symptom of dry eye disease involves reduced tear production, which leads to red eyes, burning, and severe discomfort; cases can range from uncomfortable to severely debilitating (21). Only relatively ineffective palliative treatments are available (e.g., eye drops, pore plugs), and the mechanisms that cause dry eye disease (DED) are unknown (22).

LF is one of the most important proteins in the aqueous layer, as it helps to stabilize and maintain film layers (23). Changing the lactoferrin properties has resulted in changes in the physical properties of the layer sequence (23). The concentration of Lactoferrin has been shown to decrease inflammatory ocular diseases such as dry eye disease (24). This can be potentially troublesome, as the aqueous layer mainly acts to shield the eye surface from infection. As a result, lactoferrin can be used as a biomarker to detect DED. LF is known as a host defense protein, and it has multifunction anti-inflammatory and antimicrobial properties. LF is produced under inflammatory conditions by both the epithelial cells and neutrophils. Lactoferrin was used as a biomarker for type 2 diabetes (25–27) and dry eye disease (24, 28).

In regard to evaluating inflammatory biomarkers in diabetic ocular disorders, blood is a more effective predictor of various body diseases than ocular tear fluid. A blood test accurately and consistently represents inflammation, whereas eye tear analysis is impacted by ocular factors. Routine blood testing is an excellent first step in monitoring prospective diabetes complications and improving our understanding of diagnostic and prognostic biomarkers.

Hyperglycemia leads to a neutrophil dysfunction that affects LF production and often occurs in type 2 diabetes patients (25). Numerous prior research studies have demonstrated the relationship between neutrophils and inflammatory diseases, emphasizing the use of the neutrophil to lymphocyte ratio (NLR) as a predictor for inflammatory conditions, including diabetes and DED (29–33). Few studies have demonstrated the relationship between lactoferrin and inflammatory diseases such as type 2 diabetes (34–36).

T2D is one of the most prevalent causes of mortality worldwide due to its link to a spectrum of severe microvascular and macrovascular complications. It is necessary to monitor T2D regularly to manage these complications by utilizing routine blood biomarker tests. The HbA1C biomarker is a main test for assessing long-term blood sugar management. Another important inflammatory biomarker is CRP; a routine blood test employed to assess and manage systemic inflammation (29, 37). Similarly, a complete blood count provides a biomarker for systemic inflammation for T2D, including neutrophil levels (32, 38), and lymphocyte counts (29, 39–42).

Among protein biomarkers, ALB is an abundant body protein essential for indicating insulin deficiency and serves as an inflammatory predictor (43, 44). LF is an iron-binding glycoprotein primarily secreted by neutrophils and recognized as an acute-phase protein; it is a biomarker for inflammatory diseases such as T2D (17, 45). Additionally, there are other inflammatory biomarkers derived from routine blood tests, such as the NLR (39, 46). This ratio has emerged as a reliable predictor of systemic inflammation, insulin resistance, and various complications related to T2D. Furthermore, plasma lactoferrin to neutrophil ratio (LFNR) provides a more comprehensive and nuanced insight into intricate systemic inflammation (45, 47, 48).

The systematic routine blood biomarker tests are essential for the formulation of therapeutic management plans and the prevention of the progression of complications associated with diabetes. To gain insight into the relationship between the innate immune system and metabolic disease of lactoferrin, we aimed to investigate the correlation between plasma lactoferrin and other inflammatory biomarkers, such as LFNR and HbA1C in type 2 diabetes with dry eye disease (T2D-DED) in a Case–Control human study.

## 2 Methods

### 2.1 Study population and sample size

This study was a retrospective and prospective case–control design with a consecutive sampling technique. It involved collecting data collected between Sept 29, 2021, and Aug 25, 2022. The required sample size calculated based on the prevalence of type 2 diabetes with dry eye disease from previous study (17.5%) (49). Due to the low sample size, all patients within the assigned period were included. Assuming that the number of populations will be approximately 2000 individuals, the minimum sample size will be 196 patients.

The study included a total of 67 participants divided into two groups: the T2D-DED patients as the study group (*n* = 41) and healthy individuals as the control group (*n* = 26) recruited from the Diabetes Center outpatient clinics at King Abdulaziz Medical City, in Jeddah, Saudi Arabia. It was challenging to find healthy individual volunteers of similar age to the T2D-DED group. Moreover, healthy individual volunteers were available at younger ages. This study occurred under the exceptional circumstances of the post-COVID-19 pandemic in 2021, which impacted participant recruitment. Here, convenience sampling was used, which is necessary given the limitations restricting the number of participants. Nevertheless, the robust data collected offers valuable insights into T2D-DED. The consecutive sampling technique aimed to include every accessible subject meeting the criteria and is considered a strong form of non-probability sampling for minimizing selection bias compared to pure convenience sampling. However, unprecedented logistical challenges in patient recruitment and reliance on consenting individuals resulted in numerical disparity. Despite this, the consecutive recruitment ensured that all eligible individuals were included, maximizing our sample’s comprehensiveness within practical constraints.

### 2.2 Data collection retrospectively

Physicians evaluated the diagnostic disease indices. Plasma samples were collected and analyzed for routine biomarker tests in the hospital. Patient demographic data (age and gender) and laboratory routine blood biomarkers results, including neutrophils, lymphocytes, CRP, HbA1C, and ALB data, were collected retrospectively from the Bestcare hospital information system.

### 2.3 Data collection prospectively

Following routine blood tests at the hospital, the collected plasma samples were analyzed for lactoferrin concentration using an in-house indirect ELISA method.

### 2.4 Plasma lactoferrin analysis using indirect ELISA

The blood samples were collected from patients in EDTA tubes before they were tested in the laboratory. Plasma LF concentrations were measured and analyzed using an in-house indirect ELISA method reported in 2024 by Alhalwani (27). This method involves utilizing a triplicate sample of 1:5 dilutions of the individual plasma sample in carbonate buffer, pH 9.6 (50 μL per well), followed by incubation at 4°C overnight. The wells are to be washed with one-fold Phosphate-Buffered Saline, pH 7.4 (PBS) and 0.05% Tween (2×200 μL per well), with this procedure being repeated after each incubation. Subsequently, the plate was blocked using PBS and 3% BSA (200 μL per well) and incubated at room temperature for a duration of 2 h. A primary antibody, specifically a 1:1000 dilution of Biotinylated rabbit polyclonal anti-human lactoferrin antibody in dilution buffer (50 μL per well), was then introduced to each well and incubated for 1 h at room temperature. Following this, a 1:10,000 dilution of Streptavidin Horseradish peroxidase-labeled secondary antibody in dilution buffer (50 μL per well) was added to each well and incubated for 1 h at room temperature. Subsequently, 1-step ultra 3,3′,5,5′-tetramethylbenzidine substrate (100 μL per well) was added, and the incubation continued at room temperature until the color exhibited a blue hue. Finally, the reaction was halted after 10 min by the addition of 0.5 M sulfuric acid (100 μL per well) with vigorous shaking, resulting in a yellow coloration. The optical absorbance density (OD) at 450 nm was measured using a plate reader (BioTek-SYNERGY HT), with the reference absorbance at 620 nm subtracted from the 450 nm value. To determine lactoferrin concentrations in the samples, a standard calibration curve, established with purified human milk lactoferrin, spanned concentrations ranging from 0.03 μg mL^−1^ to 2.00 μg mL^−1^ (27).

### 2.5 Statistical analysis

PRISM software (GraphPad Inc., San Diego, CA, USA) processed and analyzed the data. Categorical variables were expressed as percentages and integers. Mean and standard deviation (Mean ± SD) were used to present the parametric data.

A normality test, the Shapiro–Wilk test, was performed for the collected demographic and laboratory finding data, which were not normally distributed (*p* = 0.0001). For numerical data (laboratory findings and age), the Mann–Whitney test was used for non-normally distributed data to determine significant differences between the two groups, while the unpaired t-test was applied to normally distributed values. For categorical data (gender), the Pearson’s Chi-Square test was utilized. The correlations between LF and LFNR were examined between both groups using Spearman’s correlation tests for the non-normally distributed data and Pearson’s correlation tests for the normally distributed data between LF and HbA1C.

### 2.6 The neutrophil-to-lymphocyte ratio and the plasma lactoferrin to neutrophil ratio calculations

The plasma lactoferrin to neutrophil ratio (LFNR) is determined by dividing the plasma lactoferrin level by the neutrophil count (50). The neutrophil-to-lymphocyte ratio (NLR) was calculated as the neutrophil count divided by the lymphocyte count (51).

### 2.7 Ethical considerations

The Institutional Review Board (IRB) approved the study at King Abdullah International Medical Research Centre (IRB SP20/280/J). As part of the study, which was prospective to collect patient blood samples, it was conducted with patients’ written informed consent. All patients provided their consent following the Helsinki Declaration guidelines.

## 3 Results

### 3.1 Demographic and laboratory finding

The age mean ± SD of T2D-DED patients was 52.10 ± 15.00 years, with statistically significant differences between groups (*p* = 0.0001). The T2D-DED patients have more females than males, 51.20 and 48.70%, respectively, with statistically significant differences in the gender distribution in the groups (*p* = 0.031), as shown in Table 1.

**Table 1:** Comparison of demographic data and laboratory findings between T2D-DED patients and healthy individuals.

Parameter	T2D-DED	Healthy	*p*-value *
Age (Years)	(52.1 ± 15.00)	(29.3 ± 7.40)	**0.0001**
Gender (%)	Male (48.70%)	Male (23.07%)	**0.031**
	Female (51.2%)	Female (76.90%)	
CRP (mgmL^−1^)	6.88 ± 9.20	0.95 ± 0.92	**0.017**
ALB	42.7 ± 7.90	42.60 ± 11.80	0.317
Neutrophil count × 10^9^	9.10 ± 35.80	3.52 ± 1.45	0.392
lymphocytes count × 10^9^	2.90 ± 2.10	2.85 ± 2.87	0.076
LF (μgmL^−1^)	1.10 ± 1.0	0.5 ± 0.40	**0.016**
LFNR	0.28 ± 0.32	0.09 ± 0.10	**0.008**
NLR	1.40 ± 1.06	1.64 ± 0.89	0.120
HbA1C (%)	7.49 + 1.37	5.21 + 0.26	**0.0001**

*Mann–Whitney *U* test.

**A significance level of *p*-value < 0.05 was adopted.

Table 1 shows the laboratory findings results between T2D-DED patients versus healthy individuals. CRP levels were higher (6.88 ± 9.20) in T2D-DED group than in healthy group (0.95 ± 0.92), with a statistically significant difference between groups, *p* = 0.017. ALB levels were mainly similar in T2D-DED and healthy (42.70 ± 7.90 and 42.60 ± 11.80, respectively), with insignificant differences between groups (*p* = 0.317). Meanwhile, T2D-DED has a higher neutrophil count (9.10 ± 35.80) than healthy (3.52 ± 1.45), with an insignificant difference between groups (*p* = 0.392). The lymphocyte counts were mainly similar in T2D-DED and healthy (2.90 ± 2.10 and 2.85 ± 2.87, respectively) with negligible difference between groups (*p* = 0.008). LF level was higher in T2D-DED group (1.10 ± 1.03) than healthy group (0.50 ± 0.40), with a statistically significant difference between groups *p =* 0.016. The mean of LFNR level was higher in the T2D-DED group (0.28 ± 0.32) than the healthy group (0.09 ± 0.10), with statistically significant differences between groups, *p* = 0.0079. The means of NLR levels were different in T2D-DED group versus healthy group, (1.40 ± 1.06 and 1.64 ± 0.89, respectively), with statistically insignificant differences between groups *p* = 0.120. The mean HbA1C level was higher in T2D-DED group (7.49 ± 1.37) than healthy group (5.21 ± 0.26), with a significant difference between groups, *p* = 0.0001.

### 3.2 Correlation analysis

**Figure 1:**
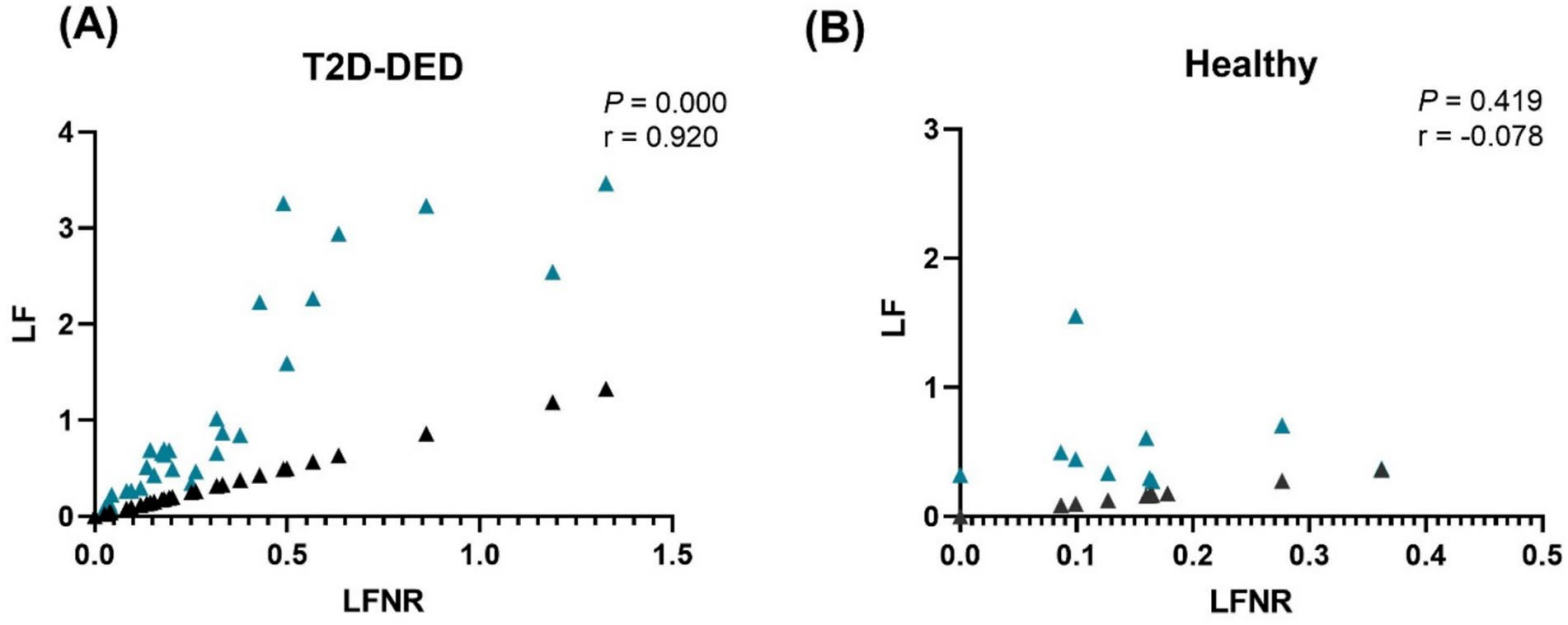
Spearman’s correlation analysis of LF (blue triangle) with LFNR (black triangle) in **(A)** T2D-DED patients and **(B)** Healthy individuals.

Spearman’s correlation analysis applied to our study groups between LF and LFNR revealed a strongly positive and statistically significant difference correlation in T2D-DED patients (*p* = 0.0001; *r* = 0.920) (Figure 1A); compared with healthy individuals, LF and LFNR were negatively correlated and not statistically significant difference correlation (*p* = 0.419; *r* = −0.078). (Figure 1B).

**Figure 2:**
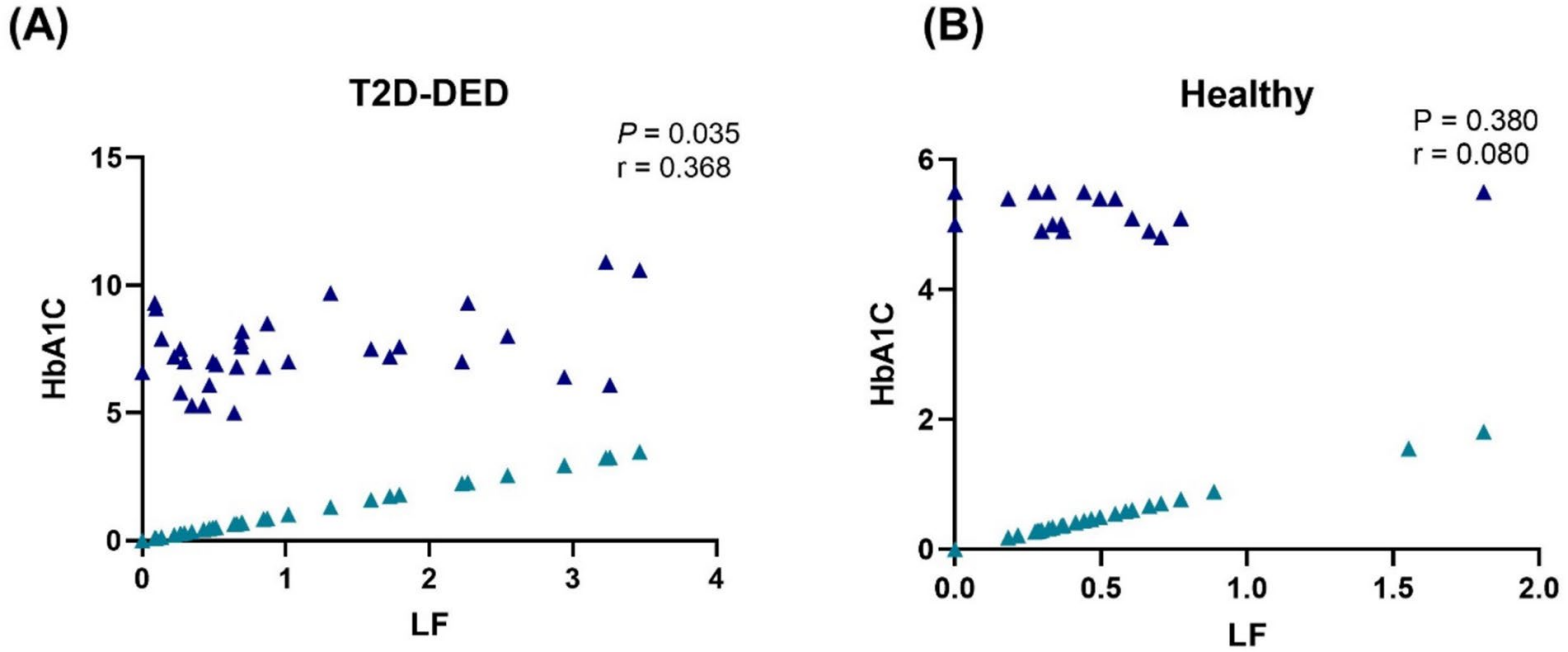
Pearson’s correlation analysis of HbA1C (black triangle) with LF (blue triangle) in **(A)** T2D-DED patients and **(B)** Healthy individuals.

A Pearson’s correlation was performed between HbA1C and LF and found a weak positive with a statistically significant difference correlation (*p* = 0.035; *r* = 0.368) in the T2D-DED patients (Figure 2A); however, weak positive and not statistically significant difference correlation (*p* = 0.380; *r* = 0.080) in healthy individuals (Figure 2B).

## 4 Discussion

### 4.1 Comparison of demographic data

Lactoferrin is a multifunctional protein that responds to multiple pathophysiological changes in the Human body. Thus, this study is an exploratory study and intends to evaluate the correlation between plasma lactoferrin and other routine blood biomarkers in type 2 diabetic patients with dry eye disease compared to healthy individuals, as studies in this manner are limited. Few studies have investigated the link between blood inflammation and blood diabetes biomarkers in type 2 diabetes and dry eye condition (29, 49, 52, 53). A study by Vorland reviewed the antimicrobial activity of lactoferrin based on protein properties (54). Another study by Lepanto et al. reported the anti-inflammation activity of LF for infectious conditions such as septic (16). Moreover, Lepanto et al. reported non-infectious conditions such as type 2 diabetes (16). Also, lactoferrin has potential therapeutic including antidiabetic effects, as evidenced in a recent randomized clinical study for type 2 diabetes in children randomly enrolled and treated with camel LF, which showed improved HbA1C and other diabetes-related parameters such as body mass index (15).

In the demographic results, it was found that T2D-DED is more common in females than male diabetic patients, which is similar to previous studies for type 2 diabetes patients (55, 56) and dry eye disease patients (57, 58). The mean age of patients with T2D-DED was classified as middle-aged. Although the occurrence of older individuals has been reported in previous studies related to type 2 diabetes (55) and dry eye disease (59, 60). Here, the findings indicate that a variety of dry eye diseases occur across a broad spectrum of adult ages (61), alongside type 2 diabetes (62).

This study identifies an age discrepancy between the control group and the study group. However, age has no influence on plasma lactoferrin levels in healthy individuals. Antonsen et al. found that plasma lactoferrin levels in healthy adults are influenced by factors other than age or gender, and levels fluctuate significantly (63). This phenomenon arises from the fact that lactoferrin levels are influenced by various factors beyond age, such as inflammation resulting from diseases and infections, encompassing both viral and bacterial agents (64).

### 4.2 Comparison of laboratory findings

The mean CRP in T2D-DED patients was higher than that of healthy individuals, which matches previous study according to Nehring SM et al. (37). Moreover, Alhalwani et al. showed matched CRP results in type 2 diabetes with dry eye disease patients (29). Furthermore, ALB levels were low in T2D-DED patients, as previously demonstrated in numerous diabetes studies, compared to non-diabetic patients, because insulin deficiency is involved in reducing ALB synthesis in the liver (43, 44). The serum ALB in this study was independently demonstrated to be related to T2D-DED patients compared to healthy individuals.

This study focused on two types of white blood cell counts that play a significant role in inflammation: lymphocyte and neutrophil counts. This study tested neutrophils due to their direct relationship to acute inflammation in expressing lactoferrin (65). In T2D-DED patients, neutrophil levels were higher than those in healthy individuals. Previous studies have linked increased neutrophil levels to various diabetic complications, driven by the chronic inflammatory processes of diabetes (38). Additionally, neutrophil counts were found to increase in T2D-DED patients, which is aligned with the increase in CRP levels. A previous study showed that increased neutrophil count was linked to increased CRP concentration in diabetic patients (32).

This study examined lymphocytes owing to their role in the adaptive immune response, which regulates and resolves inflammation (66). The lymphocyte counts were decreased in T2D-DED; this finding matches previous studies that suggested decreased lymphocyte counts and increased neutrophil counts in diabetes patients (39, 40). Lymphocyte counts in patients with DED vary due to the distinct immune responses triggered by different types of infections, which affect specific lymphocyte subsets. Additionally, the inflammation stage also plays a role, with lymphocyte counts fluctuating from acute phases to chronic states (41, 42).

Lactoferrin levels are elevated in patients with T2D-DED, as corroborated by prior research indicating that lactoferrin serves as a biomarker for T2D (25–27) and DED (24, 28).

Alhalwani (2021), recent reviews showcase lactoferrin’s helpful role in inflammation and its connection with neutrophil dysfunction, especially when we look at it through the lens of type 2 diabetes (25).

Here, the mean of NLR falls within the normal range for T2D-DED and healthy groups, and this is due to the variability of NLR, which is primarily associated with inheritance (67). The serum NLR in this study was independently demonstrated to be related to T2D-DED patients compared to healthy individuals. Alhalwani (2021), recent reviews showcase lactoferrin’s helpful role in inflammation and its connection with neutrophil dysfunction, especially when we look at it through the lens of type 2 diabetes (25). This study investigates the relationship between LF and neutrophils using the LFNR calculated biomarker. The mean LFNR increases in patients with T2D-DED, indicating a rise in systemic inflammation, as supported by previous studies (45, 47, 48).

### 4.3 Correlation analysis of lactoferrin with lactoferrin to neutrophil ratio

Fewer studies have reported results on understanding the role of plasma LF in type 2 diabetes patients with dry eye disease (34–36). In this study, plasma LF is increased in T2D-DED patients compared to healthy individuals. This finding is supported by normal plasma lactoferrin (26), lower than in diabetes patients (27). Here, the result agreed with Abdulkader et al., who showed that HbA1C and LF were increased in patients with T2D versus healthy control (26). Likewise, LFNR is increased in T2D-DED patients compared to healthy individuals, and shown before that LFNR increases in inflammatory episodes, as reported by Rosenmund et al., and increased in lactoferrin and neutrophils ratio in septicaemic patients (68).

Compared with previous studies, the NLR increased with the increase of HbA1C in diabetic patients with dry eye disease (29). According to a survey by Irene Vinagre et al., there is a positive correlation between levels of glycosylated hemoglobin A1c and Inflammatory biomarkers (69). This study also found that diabetes patients have higher levels of CRP and albumin than healthy individuals.

### 4.4 Correlation analysis of glycosylated haemoglobin A1c with lactoferrin

Moreover, since this study attempted to explore the association between plasma lactoferrin and other inflammatory indicators in T2D-DED patients, to our knowledge, there were no other studies with a similar population that were found to compare the findings with plasma lactoferrin concentration, and the correlation applied between LF and LFNR. In T2D-DED patients, a strongly positive and significant correlation was revealed. This finding matches a previous study by Rosenmund et al., which reported a positive correlation between lactoferrin and neutrophils in myocardial infarction, septicemia, and liver cirrhosis (68). In contrast to healthy groups, the lactoferrin LF and LFNR were negatively correlated and statistically insignificant. When HbA1C and LF were correlated, a weak positive but significant association was detected in the T2D-DED group but not in the healthy group. A recently reported study by Abdulkader et al. contrasts with this study, which shows an insignificant negative correlation between HbA1C and LF in patients with T2D-DED versus healthy controls (26).

These findings suggest that monitoring inflammation to identify T2D patients at higher risk of developing DED may be critical for slowing its progression and protecting the ocular surface from permanent damage.

## 5 Conclusion

The findings of this study highlight the potential utility of LFNR as an inflammatory biomarker alongside standard routine blood biomarkers CRP and HbA1C in diabetic patients with ocular complications. The significant increase of LFNR has promise for guiding T2D complication decision making, as predicting DED. The significant positive correlations between LF and HbA1C, as well as LF and LFNR, in T2D-DED comorbidities differed from those in healthy individuals, demonstrating that the potential prognostic relevance of these indicators depends on the presence or absence of disease. LF and HbA1C are promising biomarkers for the prognosis of T2D-DED patients. This investigation will shed new light on the significance of LF in diagnosing T2D-DED patients. LFNR is particularly advantageous given that it is cost-effective and easily calculated from blood count data routinely and serially monitored in patients with T2D-DED. Additional research is justified to better understand if routine observation of LFNR in research and clinical practice could beneficially impact the care of patients with T2D-DED.

### 5.1 Limitations and future work

Only plasma samples were available for healthy volunteers in the adult population, which prevented age-matched comparisons. Similarly, the study’s design was not sufficiently optimized to show the link between LF and other inflammatory indicators; an additional cohort study is required to conclude the impact of LF status on these T2D patients. Additionally, the unforeseen logistical challenges following the COVID-19 pandemic in 2021 and this study’s dependence on convenience sampling made it impossible to secure an equal number of participants in the healthy control and T2D-DED groups, without age-matched samples. Further research with larger and more diverse populations is needed to validate these results. Additional studies are required to confirm the utility of these values in measuring T2D-DED severity and to identify the cut-off values.

## Data Availability

The raw data supporting the conclusions of this article will be made available by the authors, without undue reservation.
